# Sudden upper airway obstruction during catatonia treatment: A case of nasogastric tube syndrome

**DOI:** 10.1002/pcn5.70233

**Published:** 2025-11-13

**Authors:** Kota Mukasa, Masaya Mashimoto, Yasunori Nakata, Fumihiko Sato, Hiromi Chiba, Motohiro Ozone

**Affiliations:** ^1^ Department of Neuropsychiatry Kurume University School of Medicine Kurume‐city Fukuoka Japan; ^2^ Department of Otolaryngology‐Head and Neck Surgery Kurume University School of Medicine Kurume‐city Fukuoka Japan

**Keywords:** catatonia, dementia with Lewy bodies, electroconvulsive therapy, nasogastric tube syndrome, upper airway obstruction

## Abstract

**Background:**

Nasogastric tube syndrome (NGTS) is a rare yet potentially life‐threatening complication caused by prolonged compression of the laryngeal structures by a nasogastric tube, resulting in bilateral vocal fold paralysis and acute upper airway obstruction. While NGTS has been reported in patients requiring enteral feeding due to conditions such as stroke or impaired consciousness, no cases during the treatment of catatonia have been documented. NGTS remains underrecognized despite common nasogastric use in catatonia.

**Case Presentation:**

The patient was a 66‐year‐old woman with probable dementia with Lewy bodies who presented with catatonia characterized by psychomotor retardation. Due to impaired oral intake, a nasogastric tube was inserted for nutritional support. On the 38th day after the tube was inserted, she gradually developed stridor and worsening respiratory distress, followed by paradoxical breathing. Laryngoscopy revealed bilateral abductor vocal fold paralysis accompanied by marked arytenoid edema, and an emergency tracheostomy was performed. CT imaging confirmed arytenoid edema, while brain and cervical imaging revealed no evidence of central or peripheral lesions affecting the vagus or recurrent laryngeal nerves. Based on the clinical course and findings, a diagnosis of NGTS was made. Following removal of the nasogastric tube, vocal fold mobility gradually returned to normal. Her catatonic symptoms improved significantly after a course of electroconvulsive therapy.

**Conclusion:**

This case highlights the potential severity of NGTS in psychiatric settings. In patients with catatonia, who are often unable to communicate their symptoms, careful clinical monitoring is essential. When upper airway symptoms such as stridor or hoarseness arise during nasogastric feeding, NGTS should be considered as a possible cause. A multidisciplinary approach—including prompt consultation with otolaryngology—is crucial to prevent serious complications.

## BACKGROUND

NGTS is a rare complication characterized by bilateral vocal fold abductor paralysis caused by chronic mechanical stimulation from a nasogastric tube.^1^ The underlying mechanism is thought to involve localized ulceration or edema of the arytenoid or epiglottic regions, leading to bilateral vocal fold paralysis and resulting in upper airway obstruction characterized by stridor, dyspnea, and hoarseness. The condition can be fatal if not promptly recognized and treated. Reported risk factors include older age, malnutrition, neurological disorders, impaired swallowing reflex, and the use of large‐bore nasogastric tubes.^2^, ^3^, ^4^

NGTS has primarily been reported in patients with stroke, trauma, postoperative conditions, or impaired consciousness.^4^ However, to our knowledge, no cases have been reported during the treatment of catatonia.

Catatonia is a neuropsychiatric syndrome that may be secondary to psychiatric, neurological, or medical conditions, and is characterized by symptoms such as mutism, immobility, posturing, negativism, and psychomotor agitation.^5^ Because oral intake is often impaired, enteral nutrition is commonly required in patients with catatonia.^6^, ^7^ However, the risk of NGTS is rarely addressed in this context.

Here, we present a case of NGTS that occurred during the treatment of catatonia in a patient with probable dementia with Lewy bodies (DLB). Written informed consent was obtained from the patient's husband.

## CASE PRESENTATION

A 66‐year‐old Japanese woman, 140 cm in height and weighing 35 kg, with a history of cerebral infarction, type 2 diabetes mellitus, hypertension, dyslipidemia, hyperuricemia, and osteoporosis, was admitted for the treatment of catatonia. She had no family history of psychiatric illness. The patient had experienced gradually worsening memory impairment over several months prior to hospitalization. The patient developed visual hallucinations, Capgras syndrome, and bradykinesia. As her condition progressed, she began exhibiting disrobing behavior, agitation, auditory hallucinations, and incoherent speech, which led to hospitalization at Hospital A. Although initially agitated, she soon became hypoactive and refused oral intake. A 15 French polyvinyl chloride nasogastric tube was inserted and secured at 55 cm from the tip at the nostril 14 days before her transfer to our hospital for electroconvulsive therapy (ECT), approximately two months after her admission to Hospital A. The portion of the nasogastric tube protruding from the nostril was looped along the nostril and secured to the cheek with tape. On hospital Day 4, the nasogastric tube was exchanged for a 12 French polyvinyl chloride tube, with the fixation method and insertion depth kept the same as before.

Upon transfer to our hospital, the Bush–Francis Catatonia Rating Scale score was 28, with prominent features including mutism, immobility, posturing, negativism, and rigidity. A diagnosis of catatonia was made based on the DSM‐5 criteria.^5^ Contractures were also observed in the wrists and ankles.

Laboratory tests showed normal liver, renal, and thyroid function. Creatine kinase was 25 U/L, white blood cell count was 5900/μL, and C‐reactive protein was 0.37 mg/dL. Cerebrospinal fluid analysis revealed a protein level of 54 mg/dL, glucose 83 mg/dL, and a cell count of less than 1/μL, with no signs of inflammation. Brain MRI revealed small subacute infarcts and chronic lacunar infarcts. Dopamine transporter single‐photon emission computed tomography revealed reduced specific binding ratios in the bilateral striata (right: 2.77; left: 3.11; average: 2.94). Electroencephalography demonstrated a background rhythm of approximately 8 Hz without epileptiform discharges. These findings were insufficient to account for the clinical symptoms based on either small infarcts or encephalitis.

Considering clinical symptoms, including visual hallucinations, parkinsonism, and reduced dopamine transporter uptake, the patient met the criteria for probable DLB.^8^ Given the presence of cerebral infarcts, electroconvulsive therapy (ECT) was initiated on a planned, non‐emergent basis.

**Figure 1:**
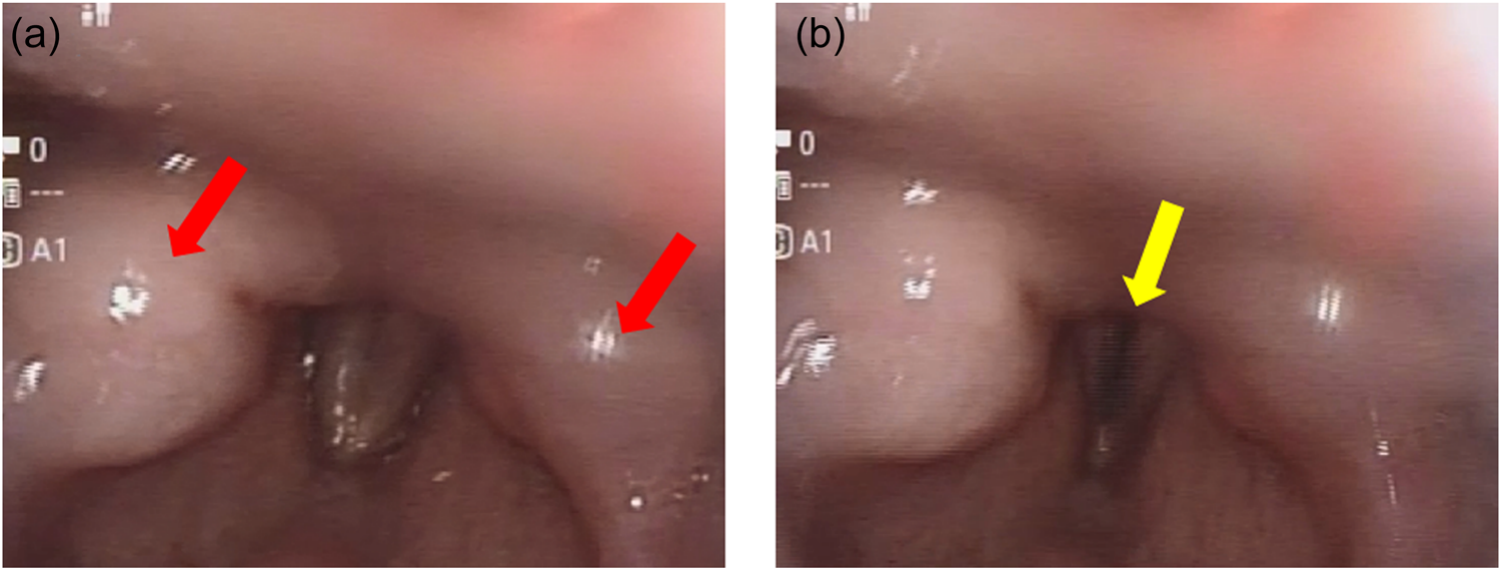
Laryngoscopic findings. a (exhalation): Marked edematous swelling is observed in both arytenoid regions, as indicated by red arrows. The edema is predominantly localized to the arytenoids, resulting in narrowing of the laryngeal inlet. b (inhalation): Bilateral abductor vocal fold paralysis is noted, with only a narrow, slit‐like glottic opening, as indicated by yellow arrows.

On hospital Day 24, which was the 38th day after nasogastric tube insertion, the patient developed intermittent stridor around noon. The patient exhibited mutism due to catatonia, and complaints such as dyspnea or throat pain were not reported. By 4:00 p.m., her oxygen saturation had declined following an episode of vomiting, although chest radiography revealed no pulmonary infiltrates. Despite oxygen supplementation, her respiratory status continued to deteriorate, and paradoxical breathing was observed. At 6:00 p.m., the patient was referred to an otolaryngologist. At 6:35 p.m., a specialist‐performed laryngoscopy demonstrated bilateral vocal fold abduction paralysis with a narrow, slit‐like glottic opening and marked edematous changes in both arytenoid regions. (Figure 1). Due to worsening hypoxemia, an emergency tracheostomy was performed, resulting in immediate improvement in oxygenation.

**Figure 2:**
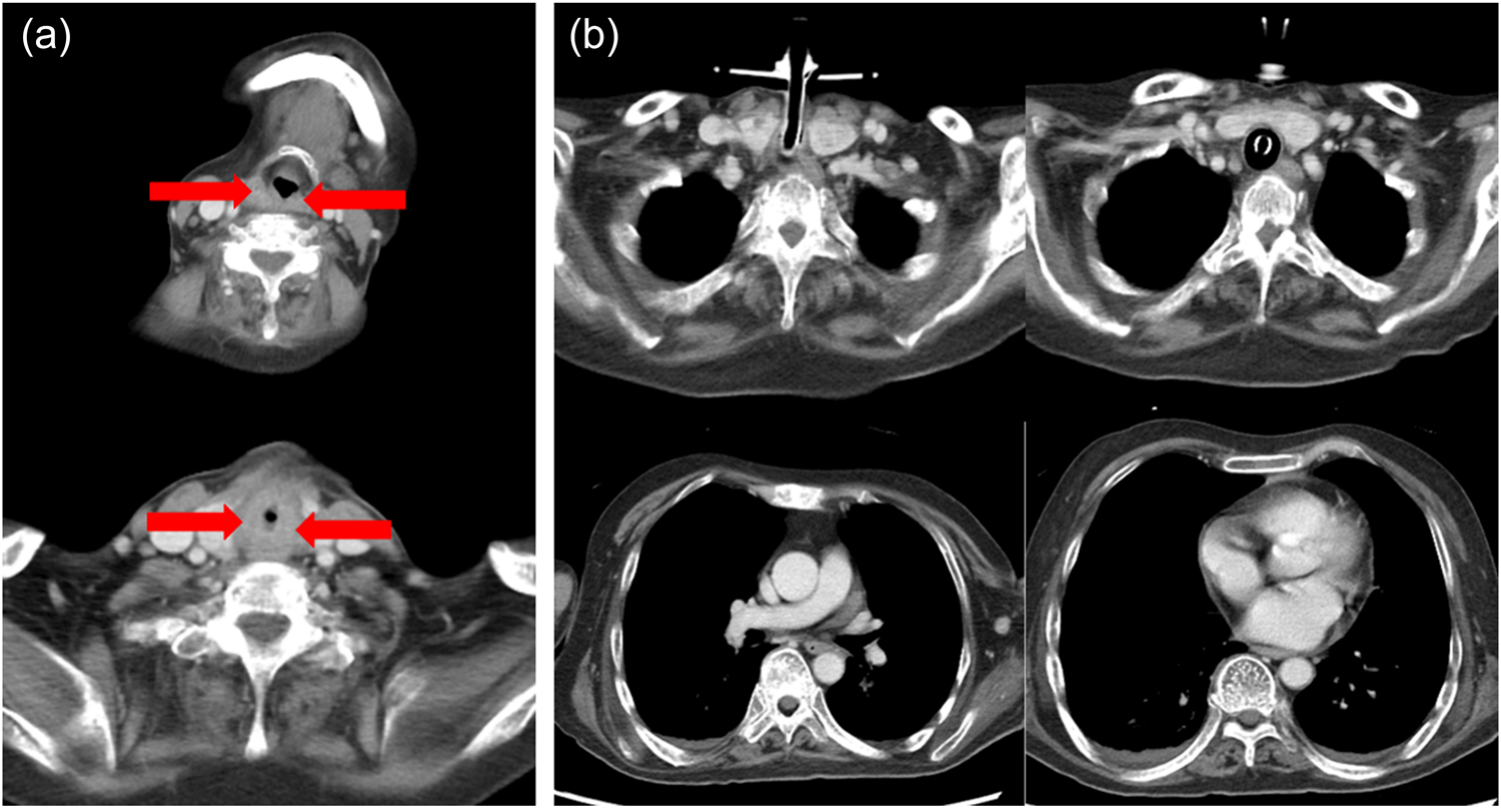
Contrast‐enhanced computed tomography. (a) The nasogastric tube is removed. Marked edema is observed in the arytenoid region, as indicated by the red arrow. (b) No structural abnormalities are identified from the neck to the chest that could explain the bilateral vocal fold paralysis.

Contrast‐enhanced computed tomography revealed edema involving the arytenoid region, aryepiglottic folds, posterior cricoid cartilage, and posterior pharyngeal wall (Figure 2). Brain and cervical imaging showed no central or peripheral lesions that could account for the bilateral vocal fold paralysis.

There was no history of trauma or surgery, and the brain MRI revealed no lesions that could account for bilateral vocal fold paralysis. Furthermore, contrast‐enhanced CT showed no neoplastic lesions or abscess formation involving the vagus or recurrent laryngeal nerves, and blood tests demonstrated no elevation of inflammatory markers. Taken together, surgical, neoplastic, radiation‐induced, traumatic, central, cardiovascular, and infectious causes were considered unlikely, and NGTS was most consistent with the patient's clinical history. The nasogastric tube was removed, leading to the gradual recovery of vocal fold mobility. By hospital Day 74, mobility had fully returned to normal, and the tracheostomy was subsequently closed. Twelve sessions of ECT were administered following tracheostomy, resulting in improvement of catatonic symptoms. The patient became able to follow simple commands and engage in short verbal interactions. However, a formal cognitive assessment could not be conducted due to impaired comprehension. Her swallowing function improved, enabling the resumption of oral intake. Delusional beliefs and visual hallucinations persisted, and joint mobility remained restricted due to contractures. She was subsequently transferred back to Hospital A for further care.

## DISCUSSION

This case illustrates the development of NGTS in a catatonic patient with probable DLB. The patient experienced life‐threatening upper airway obstruction 38 days after nasogastric tube insertion. NGTS was diagnosed based on the presence of bilateral vocal fold abduction paralysis, laryngeal edema, and clinical improvement following tube removal.

### Diagnosis of NGTS

The diagnosis of NGTS is essentially clinical and is frequently established by exclusion. The reported causes of bilateral vocal fold paralysis include surgical (61.3%), neoplastic (17.5%), idiopathic (10.3%), traumatic (1.5%), central (4.7%), cardiovascular (2%), radiation‐induced (1.5%), and inflammatory (1%) etiologies.^9^ This case had no history of trauma or surgery, and the brain MRI revealed no lesions that could account for bilateral vocal fold paralysis. Furthermore, contrast‐enhanced CT showed no neoplastic lesions or abscess formation involving the vagus or recurrent laryngeal nerves, and blood tests demonstrated no elevation of inflammatory markers. Taken together, surgical, neoplastic, radiation‐induced, traumatic, central, cardiovascular, and infectious causes were considered unlikely, and NGTS was most consistent with the patient's clinical history.

### NGTS Pathophysiology and Risk Factors

NGTS is thought to result from prolonged pressure of the tube on the arytenoid and epiglottic regions, leading to localized inflammation, edema, or recurrent laryngeal nerve palsy.^3^ Clinical manifestations include dyspnea, hoarseness, throat pain, and stridor. Reported risk factors include advanced age, malnutrition, diabetes mellitus, impaired swallowing reflex, parkinsonian syndromes, and cerebrovascular disease.^1^, ^2^ In this case, multiple risk factors for NGTS were present. The patient's catatonia prevented symptom reporting of throat pain or dyspnea, potentially leading to a delay in recognition. Previous reports have identified the use of large‐diameter nasogastric tubes as a potential contributor to the development of NGTS, with symptom improvement observed after replacing them with smaller‐caliber tubes.^10^, ^11^ Given the patient's small stature and low body weight, the 12 French polyvinyl chloride tube may have exerted excessive pressure on the laryngeal structures, contributing to the onset of the syndrome.

### DLB and Catatonia

While depression and schizophrenia are among the most common causes of catatonia, it may also occur in neurodegenerative disorders, such as dementia with Lewy bodies (DLB).^12^, ^13^ In the present case, the patient met the diagnostic criteria for probable DLB, and her catatonia was considered secondary to the underlying neurodegenerative process.

### Catatonia and Enteral Nutrition

Catatonia frequently impairs oral intake due to symptoms, such as immobility, mutism, and negativism, and early initiation of enteral feeding is recommended.^7^ However, catatonia typically requires a prolonged course of treatment, during which extended use of a nasogastric (NG) tube may become necessary. NGTS has been reported both shortly after insertion^1^, ^2^ and after several months or even years,^14^ indicating that clinicians should remain vigilant regardless of the duration of tube placement. In our case, NGTS occurred on Day 38 after tube insertion, suggesting that asymptomatic edema may have progressed insidiously and ultimately led to sudden airway obstruction.

In catatonia, patients are often unable to communicate early symptoms such as a sore throat or dyspnea, making clinical vigilance essential. The true incidence of NGTS during catatonia treatment remains unknown, highlighting the need for further epidemiological studies. In psychiatric settings—particularly those without ready access to otolaryngology—there is a concern that diagnosis may be delayed.

## LIMITATION

In this case, detailed documentation of the potential tube movement was insufficient, which limits the interpretation of the pathophysiological relationships.

## CONCLUSION

This case demonstrates that NGTS can occur even during the treatment of catatonia, emphasizing the importance of not overlooking upper airway signs and of early collaboration with otolaryngology. When long‐term nasogastric feeding is anticipated, consideration of alternative nutritional routes, selection of small‐diameter and flexible tubes, and systematic screening may contribute to the prevention of severe complications. The incidence of NGTS in patients with psychiatric disorders or catatonia remains unclear, and to the best of our knowledge, no cases have been previously reported, which underscores the novelty of this case. Furthermore, the unknown frequency highlights the need for future research.

## TAKE HOME MESSAGE

Nasogastric tube syndrome (NGTS) is a rare but potentially fatal complication that can occur even in psychiatric settings, such as during the treatment of catatonia.

In catatonic patients, who are often unable to express early symptoms, clinicians should maintain a high index of suspicion for NGTS when signs of airway compromise emerge.

Early recognition and multidisciplinary intervention, including prompt otolaryngological consultation, are essential to prevent life‐threatening outcomes.

## AUTHOR CONTRIBUTIONS

Kota Mukasa interviewed and treated the patient, performed the literature search, and wrote and revised the first draft of the manuscript. Masaya Mashimoto, Yasunori Nakata, and Fumihiko Sato interviewed and treated the patient, and rewrote and revised the manuscript. Hiromi Chiba and Motohiro Ozone contributed to writing and revising parts of the manuscript. All authors contributed to and approved the final version of the manuscript.

## CONFLICT OF INTEREST STATEMENT

The authors have no conflicts of interest to declare.

## ETHICS APPROVAL STATEMENT

This study was conducted in accordance with the principles outlined in the Declaration of Helsinki.

## PATIENT CONSENT STATEMENT

Written informed consent was obtained from the patient's husband for presentation of their clinical course.

## CLINICAL TRIAL REGISTRATION

Not applicable as this is a case report.

## Data Availability

Research data are not shared.
